# General Violence Against Women by an Intimate Partner and Co-Occurrence

**DOI:** 10.1177/26884844251390892

**Published:** 2025-10-30

**Authors:** Franciéle Marabotti Costa Leite, Bruna Venturin, Nathália Miguel Teixeira Santana, Tiffani Matos Oliveira, Tamires Paulo Ceccon, Luíza Eduarda Portes Ribeiro

**Affiliations:** ^1^ Federal University of Espírito Santo, Vitoria, Brazil.; ^2^ Federal University of Pelotas, Pelotas, Brazil.

**Keywords:** violence, intimate partner violence, cross-sectional studies

## Abstract

**Background:**

This study aims to estimate the general prevalence of violence against women and its associated factors, including co-occurrence in the municipality of Vitória, Espírito Santo.

**Methods:**

This population-based cross-sectional study was carried out in the municipality of Vitória, Espírito Santo, Brazil, with 1086 women who were interviewed in their homes. Data were analyzed in Stata 15.1. The chi-squared test of heterogeneity was used for the bivariate analysis. Poisson’s regression with robust variance estimation was used to calculate crude and adjusted prevalence ratios and their respective 95% confidence intervals.

**Results:**

Overall violence throughout life represented a total of 47.8%, 13.0% of which co-occurred with psychological, physical, and sexual violence. Eleven percent of the participants reported psychological and physical violence episodes. Women between 40 and 49 years old had a 31% higher prevalence of intimate partner violence in their lifetime than older women. Catholics had a 33% lower prevalence of violence than non-Catholics. Those who lived without a partner endured a 1.4 times greater occurrence of violence. Women with up to 8 years of schooling and belonging to the second socioeconomic tertile had higher frequencies of violence. Regarding family experiences of violence, women whose mothers had been attacked by their partners had a 32% higher prevalence of overall violence in their lives (*p* < 0.05).

**Conclusions:**

This study associated victims’ polyvictimization and some of their main behavioral and family characteristics that can increase their chances of suffering violence. It is also important that future national and international studies evaluate the co-occurrence of violence experienced by women.

## Background 

Violence against women is a complex social phenomenon and a violation of human rights, potentially causing physical, reproductive, sexual, and psychological harm, as well as affecting interpersonal relationships for generations or even a lifetime.^1^ Most cases of violence against women have intimate partners as their main perpetrators and occur indoors.^2^ Intimate partner violence (IPV) refers to any action by partners or former partners that physically, sexually, or psychologically harms victims, including physical aggression, sexual coercion, psychological abuse, and controlling behaviors.^3^

According to the World Health Organization (WHO), one in three adult women in the Americas have experienced physical and/or sexual violence, and up to 38% of women murders are committed by a male intimate partner worldwide.^4^ Gender equality was incorporated as a goal to be achieved by 2030 among the Sustainable Development Goals agreed upon by several countries. Various government spheres must eliminate all forms of violence against all women and girls and promote discussions that can minimize the negative effects of gender inequality in the community.^5^

The COVID-19 pandemic has further evinced the need to address this issue due to the increase in cases of violence after social isolation. In Brazil, 7691 women were violently killed from 2020 to 2021. Concerning Brazilian states, Espírito Santo remained in third place among those with the greatest increase in this outcome during the pandemic (22.7%), second only to Amazonas and Piauí.^6^ In the municipality of Vitória, the capital of the state of Espírito Santo, data collected in 2014 from female users of its health system showed that psychological violence was the most common form of aggression against women (25.3%), followed by physical (9.9%) and sexual violence (5.7%), a fact that corroborates other studies in the literature.^7–9^

It is also important to highlight the co-occurrence of these types of violence in women, that is, they often suffer two or more types of violence at the same time. In one Southeast Asian country, 1372 (20.70%) women reported experiencing psychological, physical, and/or sexual IPV at some point in their lives. In Pakistan, 1505 women reported experiencing one or more of the three types of IPV.^8,9^

It is also necessary to identify the various factors that have been associated with the occurrence and perpetuation of IPV, including younger age, low education, low income, “separated” or “divorced” marital status, use of alcohol and other drugs, greater number of children, acceptance of violence, and partners’ controlling behavior.^7,8,10^ Traumatic childhood experiences, such as abuse and aggression, indicate another highly relevant associated factor, which increases the probability of women entering or remaining in abusive relationships.^7,8^

Regarding its negative health consequences, IPV against women configures an important risk factor for sexually transmitted infections such as HIV, unwanted pregnancies, abortions, and greater occurrence of mental disorders (such as depression, suicide, and use of alcohol and other drugs), in addition to negatively affecting the development of children from families experiencing such violence.^11–13^

Women constitute a vulnerable population to the worsening of violence, as the deeply rooted patriarchal culture in many countries renders them susceptible to the dominance of a male figure.^4^

Women are vulnerable to male dominance in patriarchal cultures, highlighting the complexity of addressing this issue and the need for multifaceted approaches to prevent and intervene against IPV. Effective policies and programs must address individual and behavioral aspects and the structural and social issues that perpetuate violence against women.^14^ Many challenges take part in discussing this issue, including significant underreporting of data and political and cultural barriers in addressing IPV, which directly influence the development of effective public policies to prevent this outcome, highlighting the importance of discussing it in the literature.^6^

This study aims to estimate the general prevalence of violence against women, associated factors, and co-occurrence in the municipality of Vitória, Espírito Santo.

## Methods

This cross-sectional, analytical, and population-based study was carried out in the municipality of Vitória, Espírito Santo, Brazil, in 2022. The study population consisted of women aged 18 years or older who had or have had an intimate partner in the 24 months prior to the interview. The demographic characterization of the study population can be found in the article IPV against women during COVID-19.^15^ Intimate partners were defined in this study as partners, ex-partners, and/or current boyfriends as long as they had sexual relations, regardless of a formal union.

The size required for this study totaled 1100 women, considering percentages for losses, refusals, and control for confounding factors. A refusal occurred when the selected participant in the household did not agree to participate and thus was not replaced. Additionally, if the woman was not found at her household after three attempts by the interviewer, the participant would be considered a nonresponse. Multiple stages were adopted as the sampling process with census tracts as primary units. To assess the number of selected sectors, the 2010 census data were used, which reported 108,515 households in the urban area being traced. This number was divided by 100 (the number of sectors to be visited) to obtain the systematic gap, with probability proportional to the number of households and women within each sector.

Subsequently, the list was ordered by socioeconomic level, and the number 513 (between 1 and 1085) was randomly drawn using the R program, determining the first sector number. The selection of the remaining sectors (99) was made by adding the systematic gap of the initial sector (184) and continuing this process until the end of the list. After selecting the census sectors, households were randomly selected from the IBGE’s (Brazilian Institute of Geography and Statistics) online platform. For the sample size estimation, an estimated prevalence of IPV in the studied population of 50% was used to maximize the sample, with a 95% confidence level and an acceptable margin of error of 5%. For the study of risk factor associations, a 95% confidence level, 80% power, and an exposed/unexposed ratio of 1:1 were considered. To this value, 10% for losses and 30% for confounding factors were added, determining the final sample size.

The interviews were conducted in a reserved place in the drawn households, respecting interviewees’ privacy and confidentiality. The translated and validated version of the World Health Organization Violence Against Women questionnaire was used to evaluate the studied outcome (IPV during the pandemic).^16^ It tracks the occurrence of types of IPV (psychological, physical, and/or sexual), which was considered present if the interviewees answered yes to at least one item for each violence type. Overall violence was deemed present when the interviewee answered yes to at least one item of the entire questionnaire.

The independent variables of the study were age group (up to 29 years; 30–39 years; 40–49 years; 50–59 years; 60 years or older); race/skin color (White; Black; Brown); Catholic religion (no; yes); Evangelical religion (no; yes); current marital status (living without a partner; living with a partner); years of schooling (0–8 years; 9–11 years; 12 years or more); household income tertile (first tertile—poorest; second tertile; third tertile—richest). Regarding the characteristics of family and life experiences of violence, the variables were: mother had been attacked by a partner (no; yes) and sexual violence during childhood (no; yes).

The analyses were performed in Stata®, version 15.1. The descriptive analysis of IPV prevalence was performed with crude and relative frequencies. The Chi-squared test of heterogeneity was used for the bivariate analysis. Poisson’s regression with robust variance estimation was used to estimate crude and adjusted prevalence ratios (PR) and their 95% confidence intervals (95% CI). Initially, the variables associated with the outcomes under study (*p* < 0.20) were included in the model to consider possible confounding factors. The backward selection method was used to exclude variables. A significance level of 5% (*p* < 0.05) was adopted in the final model.

This research was approved by the Research Ethics Committee at the Federal University of Espírito Santo under the following opinion**:** 2.819.597.

## Results

Most women with a history of lifetime violence were under 50 years old (64.0%), self-identified as Brown (42.9%), were Evangelical (38.2%), lived with their partner (74.6%), had 12 or more years of education (45.1%), belonged to the first income tercile (lowest; 39.8%), and did not have a history of physical violence by their mother (69.6%) or sexual violence in childhood (83.4%) (data not presented in the table).

The frequency of overall violence against women by an intimate partner throughout life among the interviewees was 47.8% (95% CI: 44.8–50.8). Around 52.0% of those interviewed did not report any occurrence of violence (data not shown in the table).

**FIG. 1.**
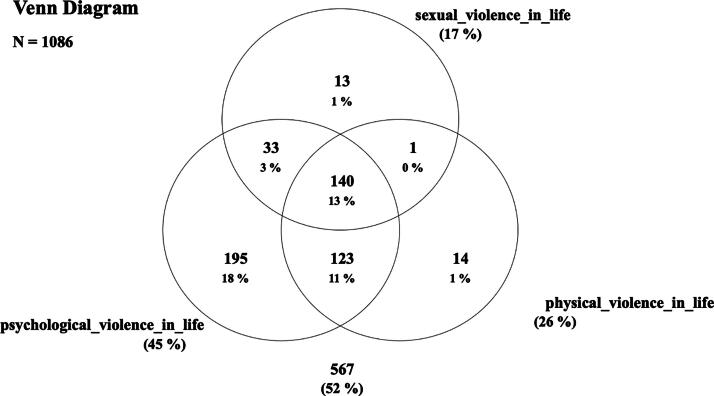
Co-occurrence of psychological, physical, and sexual violence throughout life among women living in the municipality of Vitória, Espírito Santo, Brazil (*N* = 1086).

Of the total number of interviewees (*N* = 1086), 13.0% (*N* = 140) had co-occurrence of the three types of violence (psychological, physical, and sexual) throughout their lives, and 11% (*N* = 123) reported episodes of psychological and physical violence (Fig. 1).

Table 1 shows the results of the bivariate analysis of overall violence throughout life and demographic, socioeconomic, family, and life experience characteristics. Elderly females (60 years or above) showed the lowest frequency of overall violence in life (35.1%, 95% CI: 29.6–41.1, *p* < 0.001). A higher prevalence of overall violence against women throughout life occurred among Black, non-Catholic, Evangelical, those who live without a partner, lower education levels, and those who belong to the poorest income tertile (*p* < 0.05). Regarding family and life experience, women whose mothers had been attacked by their partners and those with a history of sexual abuse in their childhood showed a higher occurrence of overall violence in their lives (*p* < 0.001) (Table 1).

**Table 1.** Prevalence of Overall Intimate Partner Violence Throughout Life According to Demographic, Socioeconomic, and Family and Life Experience Characteristics (Vitória, ES, Brazil)

Variables Demographic and socioeconomic	Overall violence throughout life		
	%	95% CI	*p* value
Age group			<0.001^a^
Up to 29 years	56.9	50.0–63.6	
30–39 years	50.0	43.2–56.8	
40–49 years	51.8	45.2–58.3	
50–59 years	48.5	41.5–55.5	
60 years or above	35.1	29.6–41.1	
Race/skin color^a^ (*N* = 1063)			0.048^a^
White	43.6	39.0–48.3	
Black	52.0	45.0–59.0	
Mixed-race	50.8	46.1–55.5	
Catholic			<0.001^a^
No	54.7	50.8–58.5	
Yes	38.3	33.9–42.8	
Evangelical			0.012^a^
No	45.0	41.4–48.7	
Yes	53.1	48.0–58.1	
Current marital status			<0.001^a^
Living with a partner	43.7	40.4–47.0	
Living without a partner	66	59.2–72.2	
Years of schooling			0.045^a^
0–8 years	53.2	46.3–60.0	
9–11 years	50.3	45.1–55.5	
12 or over	44.1	39.9–48.3	
Family income tertile (*N* = 1070)			<0.001^a^
1st tertile—poorest	57.9	52.9–62.7	
2nd tertile	44.6	39.5–49.8	
3rd tertile—richest	39.5	34.3–44.9	
Family and life experiences			
Mother has been attacked by a partner			<0.001^a^
No	42.9	39.5–46.3	
Yes	66.1	59.7–71.9	
Sexual violence in childhood			<0.001^a^
No	44.6	41.5–47.7	
Yes	74.8	66.1–81.9	

^a^ Chi-squared test of heterogeneity.

*N*, absolute frequency; %, relative frequency; 95% CI, 95% confidence interval.

Table 2 shows the crude and adjusted analysis of overall IPV according to interviewees’ demographic, socioeconomic, family, and life characteristics. After adjustment, women aged 40–49 years had a 31% higher prevalence of IPV in their lifetime than older women (PR: 1.31, 95% CI: 1.06–1.61, *p* < 0.05). Catholic women had a 33% lower prevalence of overall violence in their lifetime than non-Catholics (PR: 0.77, 95% CI: 0.67–0.89, *p* < 0.001).

**Table 2.** Crude and Adjusted Analysis Between Overall IPV Throughout Life and Women’s Demographic, Socioeconomic, and Family and Life Experience Characteristics (Vitória, ES, Brazil)

VariablesDemographic and socioeconomic	Crude analysis			Adjusted analysis^a^		
	PR	95% CI	*p* value	PR	95% CI	*p* value
Age group			0.007			0.163
Up to 29 years	1.62	1.23–2.13	0.001	1.19	0.95–1.49	0.123
30–39 years	1.42	1.07–1.89	0.014	1.22	0.98–1.53	0.071
40–49 years	1.48	1.12–1.94	0.005	1.31	1.06–1.61	0.012
50–59 years	1.38	1.04–1.84	0.028	1.23	0.98–1.54	0.071
60 years old or above	Ref.			Ref.		
Race/skin color			0.204			—
White	Ref.			—		
Black	1.19	0.94–1.52	0.148	—		
Mixed-race	1.17	0.96–1.42	0.121	—		
Catholic			<0.001			<0.001
No	Ref.			Ref.		
Yes	0.70	0.53–0.84		0.77	0.67–0.89	
Evangelical			0.070			0.556
No	Ref.			Ref.		
Yes	1.18	0.99–1.41		0.96	0.83–1.11	
Current marital status			<0.001			<0.001
Living with a partner	Ref.			Ref.		
Living without a partner	1.51	1.24–1.84		1.40	1.23–1.60	
Years of schooling			0.199			0.071
0–8 years	1.21	0.96–1.51	0.105	1.24	1.03–1.49	0.022
9–11 years	1.14	0.94–1.38	0.185	1.08	0.93–1.27	0.312
12 or over	Ref.			Ref.		
Household income tertile			0.001			<0.001
1st tertile—poorest	1.13	0.89–1.42	0.309	1.04	0.87–1.24	0.679
2nd tertile	1.47	1.18–1.82	0.001	1.32	1.12–1.55	0.001
3rd tertile—richest	Ref.			Ref.		
Family and life experiences						
Mother has been attacked by a partner			<0.001			<0.001
No	Ref.			Ref.		
Yes	1.54	1.27–1.87		1.32	1.16–1.50	
Sexual violence in childhood			<0.001			<0.001
No	Ref.			Ref.		
Yes	1.68	1.33–2.11		1.47	1.28–1.68	

^a^ Adjusted for age group, catholic, evangelical, current marital status, years of schooling, household income tertile, mother has been attacked by a partner, and sexual violence in childhood.

PR, prevalence ratio; 95% CI, 95% confidence interval.

Those who lived without a partner showed a 1.4 times greater occurrence of violence (PR: 1.40, 95% CI: 1.23–1.60, *p* < 0.01). Women with up to 8 years of schooling and belonging to the second tertile had higher frequencies of overall violence than those in the reference group, conditioning confounding factors (PR: 1.24, 95% CI: 1.03–1.49; PR: 1.32, 95% CI: 1.12–1.55, *p* < 0.05). Regarding family experiences of violence, women whose mothers had been attacked by their partner had a 32% (95% CI: 1.16–1.50) greater prevalence of overall violence in their lives (*p* < 0.05). After controlling for confounding factors, women with a history of sexual violence in childhood were almost 1.5 times more likely to be victims of overall violence in their lifetime (*p* < 0.05) (Table 2).

## Discussion

Violence against women is a human rights violation that constitutes a serious global public health problem. This is the first population-based study on violence against women in Espírito Santo to investigate the frequency of this outcome in its female population and its association with the co-occurrence of types of violence and the main risk factors for it, such as income, marital status, and education, among others.

Results showed that a significant percentage of women from Espírito Santo have already suffered IPV in their lives (47.8%). These findings exceed those of Latin America and the Caribbean (25.0%), the United States (37.3%), and Saudi Arabia (44.8%).^3,14,17^ Evaluating lifetime IPV in other Brazilian states, Espírito Santo presents similar numbers to the state of São Paulo (46.4%) and is slightly lower when compared with the state of Pernambuco (54.2%).^18^ On the other hand, the IPV reported in public and private health care services in Brazil between 2011 and 2017 was 62.4%, with Espírito Santo having the highest proportion among all other states (67.6%).^19^

These high percentages can be explained by the power relationship from the historical and social roles imposed on women, which transcend their biological characteristics.^19,20^ Moreover, culture expresses violence, being lower in egalitarian societies and more visible in predominantly male ones since the notion of masculinity is based on dominance, insensitivity, and honor, which can lead to the interpretation of violence as an inherent characteristic of men.^7,18,21,22^

At the same time, the ideal woman presupposes obedience, provides care for the house and the children, and fidelity to her partner,^21,23^ making them susceptible to the idea of possession by men, justifying obsessive jealousy and controlling behavior, in which psychological abuse configures a way of domination.^21,24^

Thus, the expression of this type of behavior in the family universe establishes gender relations in the model of hierarchical relations and normalizes and naturalizes acts of violence against women since their childhood. Most cases occur inside homes as young women stay there for longer (which often protects aggressors, contributing to maintaining women in the cycle of violence).^1,7,25–29^

In addition to victimization, results point to the co-occurrence of psychological, physical, and sexual violence throughout life. According to Owoaje et al. (2012),^30^ women, especially those of reproductive age, who have suffered physical and sexual violence are more likely to endure the co-occurrence of psychological and even sexual violence than those who have experienced no such situation.^31,32^ Based on this perspective, the various types of violence often overlap each other, in which psychological coercion and sexual abuse often follow physical violence.^17,21,33^

The highest prevalence of partner victimization was concentrated among women aged from 40 to 49 years, who showed a 31% higher prevalence than older women (PR: 1.31, 95% CI: 1.06–1.61, *p* < 0.05). Although the global IPV scenario reported by the WHO emphasizes young adult victims (15–24 years), it is important to stress that culture and life expectancy differences may justify diverse findings across countries, increasing the importance of comparing the data in this research to other Brazilian studies.^3,34^

In the state of Mato Grosso do Sul, 53.3% of victims were adult women, and in the state of Espírito Santo, 66.6% of victims were between 20 and 59 years old.^35,36^ When compared with association data, Fiorotti et al. (2023) point to the age group above 40 years old as the most often victimized (PR: 1.12, 95% CI: 1.08–1.16). It is possible that this result can be explained by the fact that younger women may have less financial autonomy, as well as a greater dependence on men when it comes to childcare.^15^

Regardless of age, women are more prone to exposure to IPV, especially under social isolation due to the COVID-19 pandemic, which has hindered case reporting and distanced victims from support networks.^3,37,38^ It is still worth highlighting the vulnerability of adult women of reproductive age to domestic violence, considering aggressors’ greater access to victims and the financial and emotional dependence due to unequal gender romantic relationships.^3^

As for aggressors, women who lived without a partner showed a higher occurrence of violence (PR: 1.40, 95% CI: 1.23–1.60, *p* < 0.01). Other studies only indicate the bond between perpetrators and victims rather than their coexistence, in which previously known perpetrators occur more often, especially partners and ex-partners.^36,38–40^ As gender inequality confers greater power to the male figure, the occurrence of violent acts inside and outside the family environment reinforces a sexist culture and perpetuates violent practices against women in relationships.^11,39^

Regarding victims’ religion, Catholic women had a 33% lower prevalence of overall violence in their lives than non-Catholics (PR: 0.77, 95% CI: 0.67–0.89, *p* < 0.001).

The literature fails to directly address Catholicism as a protective factor against violence. However, studies on victims of other non-Catholic religions (such as Evangelicals) show a higher prevalence of partner violence among these women. Evangelical women who suffer violence more naturally accept experiences of male domination, increasing their exposure to IPV.^41–43^

Women with lower schooling show a higher prevalence of violence, as in other studies in the state of Espírito Santo.^41,43^ Women who study more tend to tolerate, naturalize, and accept situations of violence by their partners less and have greater autonomy in their emotional and financial lives.^43^ Victims belonging to the second income tertile had higher frequencies of overall violence than those in the richer reference group. Both low- and high-income people may suffer from a significant prevalence of violence, showing how the worsening of violence can permeate and impact all social classes. However, lower-income women endure the greatest consequences due to their greater financial dependence.^34,43^

The association in this study between women whose mothers had been attacked by their partners and the higher prevalence of overall violence in their lives, in comparison with those without such experiences, resembles a study conducted with primary care users. It also compares with a study carried out in Pakistan, in which women who were aware of parental physical IPV showed about three times higher odds of IPV in adulthood than those who were unaware.^7,9^

Findings may also lead to reflections on the extent to which witnessing family experiences of violence in childhood configures an important marker of vulnerability to experiencing IPV. A study with 48 low- and middle-income countries found that women who witnessed IPV in childhood had more than twice the prevalence (43%) of IPV than the overall sample (21%).^43^ It also found that women with a history of sexual violence in their childhood showed a higher prevalence of IPV; results in line with a study in which victims of sexual violence in childhood had a higher prevalence of psychological and sexual IPV, respectively (*p* < 0.05).^6^ Experiences of sexual abuse in childhood are considered strong risk factors for IPV experiences and should foster appropriate interventions to interrupt this cycle of violence.^14^

These findings highlight the importance of health services monitoring women with experiences of sexual violence in childhood and adulthood to expand care, intensify guidance, and increase effective multisectoral interventions and referrals to prevent the recurrence of these outcomes. It is important to regard violence as a safety problem and a major public health issue to prepare sectors and guarantee access to services centered on the fact that IPV directly impacts the lives of women, children, and societies as a whole. Among the limitations of the study, we can include potential selection bias due to dropouts and refusals, and information bias related to the concept of violence, which may influence the accuracy of the data provided by the women and lead to an underestimation of the reported prevalence, as well as reverse causality due to the study design itself. However, the variables examined are crucial for understanding the context of violence.

This research is the first population-based study in the municipality of Vitória/ES to assess the prevalence of violence across women’s lives in its various forms, which is a significant strength and highlights the co-occurrence of different types of violence. Another aspect to be highlighted is the employment of a widely used epidemiological instrument that has been validated in Brazil to detect the occurrence of IPV, reducing the common underestimation related to the concept of violence and possible information biases. This study also minimized these biases by employing female interviewers and a private place for applying the questionnaire.

## Conclusions

This study found significant overall violence against women in the studied region, which also occurs worldwide. It associated victims’ polyvictimization with some of their main characteristics, such as lower education and income. Moreover, witnessing violent acts against their mother by their father or partner, or suffering previous violence as a child, increases the chances of women enduring such suffering. This increasingly calls for a deep understanding of violence against women, the implementation of appropriate interventions for each victim, and public policies suited to the groups most at risk. It is also important that more national and international studies be carried out on the co-occurrence of violence experienced by women.

## Data Availability

The authors declare that pertinent data and materials may be made available upon approval of the article.

## Abbreviations Used

CI: confidence intervals
COVID-19: Coronavirus Disease 2019
ES: Espírito Santo Santo
HIV: Human Immunodeficiency Virus
IBGE’s: Brazilian Institute of Geography and Statistics
IPV: Intimate partner violence
PR: adjusted prevalence ratios
SDG: Sustainable Development Goals
WHO: World Health Organization
